# Prolonging life and delaying death: The role of physicians in the context of limited intensive care resources

**DOI:** 10.1186/1747-5341-4-3

**Published:** 2009-02-12

**Authors:** Robert C McDermid, Sean M Bagshaw

**Affiliations:** 1 Division of Critical Care Medicine, University of Alberta Hospital, University of Alberta, Edmonton, Alberta, Canada

## Abstract

Critical care is in an emerging crisis of conflict between what individuals expect and the economic burden society and government are prepared to provide. The goal of critical care support is to prevent suffering and premature death by intensive therapy of reversible illnesses within a reasonable timeframe. Recently, it has become apparent that early support in an intensive care environment can improve patient outcomes. However, life support technology has advanced, allowing physicians to prolong life (and postpone death) in circumstances that were not possible in the recent past. This has been recognized by not only the medical community, but also by society at large. One corollary may be that expectations for recovery from critical illness have also become extremely high. In addition, greater numbers of patients are dying in intensive care units after having receiving prolonged durations of life-sustaining therapy. Herein lies the emerging crisis – critical care therapy must be available in a timely fashion for those who require it urgently, yet its provision is largely dependent on a finite availability of both capital and human resources. Physicians are often placed in a troubling conflict of interest by pressures to use health resources prudently while also promoting the equitable and timely access to critical care therapy. In this commentary, these issues are broadly discussed from the perspective of the individual clinician as well as that of society as a whole. The intent is to generate dialogue on the dynamic between individual clinicians navigating the complexities of how and when to use critical care support in the context of end-of-life issues, the increasing demands placed on finite critical care capacity, and the reasonable expectations of society.

## The problem

Critical care is in an emerging crisis of conflict between what individuals expect and the economic burden society/government is prepared to provide. The primary goal of advanced life support in an intensive care unit (ICU) is "to prevent unnecessary suffering and premature death by treating reversible illnesses for an appropriate period of time"[1]. The timely initiation of intensive monitoring and technological support in an ICU environment in the appropriate patient population can lead to improved clinical outcomes [2-4]. Advanced life support has also become more sophisticated, allowing physicians to prolong life in circumstances that were not possible in the recent past. In fact, one out of every six Canadians now die after support in an ICU setting [5-7]. However, while intensive care is an effective tool in the treatment of critical illness, the depiction of advanced life support and modern medical technology in the public media is often distorted, and positive outcomes are frequently overstated [8]. Consequently, societal expectations for recovery from critical illness can be unrealistically high.

The Canadian Medical Association Code of Ethics states that physicians must advocate for their patients and embrace the ethical principle of non-maleficence (Canadian Medical Association Code of Ethics, available at: ). However, physicians are often placed in a conflict of interest by pressures to use resources prudently and promote the equitable access to health care. When the demand for ICU resources exceeds available capacity, physicians are forced to triage patients, often based on subjective assessment of perceived medical benefit, or resort to other methods of rationing ICU access at the bedside. This remarkably complex and multi-faceted process impacts not only patients and physicians, but also health policy makers and society[9] These conflicting principles have the potential to compromise the safety and quality of the health system, defined by the Institute of Medicine as the "degree to which health services for individuals and populations increase the likelihood of desired health outcomes and are consistent with current professional knowledge"[10] The time-dependent effectiveness of critical care medicine and the high mortality associated with either delaying or withholding therapy [2,11-13] create tremendous challenges and polarize the consequences of the conflict, making it increasingly relevant to this specialty – use the resource immediately or withhold/deny therapy and allow the patient to die. Often there is no time for deliberation beyond a few minutes.

Currently, there is a strong perception by health care providers that ICU resources in Canada are insufficient for the demand. Since 2006, more than 150 critically ill Canadians have been emergently transferred to American hospitals due to the unavailability of Canadian ICU beds [14]. Furthermore, triage decisions are affected by capacity, and mortality is affected by the decision to permit or refuse admission to the ICU. A systematic review by Sinuff et al demonstrated that acuity of illness on admission and discharge was higher during periods of bed shortage, and that patients denied access to ICU had a three-fold increase in mortality when compared to those admitted [9]. While these data may suggest more critical care resources are urgently needed, it may also be equally argued that the existing resources need to be stewarded more judiciously since direct evidence that care is compromised by unavailability of ICU beds is limited. Sinuff et al found despite shorter lengths of ICU stay and higher acuity of illness on admission and discharge from ICU during periods of bed shortages, ICU mortality and readmission rates did not change [9]. However, additional relevant clinical endpoints, such as functional outcome or quality-adjusted life-years gained (i.e. cost-utility analysis of ICU support) were not specifically evaluated.

Despite uncertain data on outcomes, Canadian ICUs are consistently operating at over 90% capacity. Operation as such high capacity translates into reduced flexibility for accommodating new critically ill patients in need of support. Herein lays the emerging crisis – critical care treatment must be available in a timely fashion for those who require it urgently, yet its provision is largely dependent on a finite availability of both capital and human resources. Ultimately, the critical challenge remains how to provide this limited and expensive therapy for patients who will be most likely to benefit, while at the same time avoiding prolonged treatment in those patients who will not survive.

## Decision-making and therapeutic futility

The ethical principles of beneficence, non-maleficence and autonomy have shifted the practice of medicine from a predominantly paternalistic approach towards a model of shared decision-making. In critical care, decisions regarding which patients may meaningfully benefit from advanced life support are a daily challenge. The majority of Canadian families want the opportunity for discussion and input into these important decisions, but most feel that the physician has an equal or greater role than the family in end-of-life decision-making [15]. Thus, physicians must try to provide accurate timely information to the patient and family regarding prognosis for survival, morbidity and expected quality-of-life. Furthermore, broad goals of therapy must be clarified to formulate effective therapeutic plans. Ideally, this occurs through a combination of undemanding communication, a wait-and-see approach and thoughtful paternalism. Importantly, physicians must engage in this process with honesty and openness, recognizing their own biases and limitations in ability to prognosticate [8]. Yet, during bed shortages, we believe this process may be unduly prejudiced. Accordingly, decision-making on ICU support, prognostication and end-of-life care mandates guidance by high-quality evidence whenever possible.

Therapeutic futility is commonly invoked to justify either denial, limitation or withdrawal of ICU support [16]. The Society of Critical Care Medicine defines therapeutic futility as treatment that "does not accomplish its intended goal, that is, beneficial physiologic effect" [17]. The American Thoracic Society statement on Withdrawing and Withholding Life Sustaining Therapy[18] defines futility as the combination of two criteria, 1) lack of medical efficacy, as judged by the patient's physician, and 2) lack of a meaningful survival, as judged by the personal values of the patient. When a therapy is unlikely to result in survival itself, this second criterion for defining futile care becomes unnecessary. Unfortunately, the threshold for defining futility is unclear, controversial, and is often viewed differently from (and between) the critical care providers' and recipients' perspectives. The result is that discussions of therapeutic futility can be fraught with difficulties in reaching consensus between family and physician regarding the goals and direction of care [19]. Thus, at the bedside, ethical justification for withholding or the withdrawal of life support (including refusal to admit to ICU) against patients' or surrogate decision-makers' wishes is difficult without an operational definition of futility, which at present does not exist.

Similarly, non-maleficence can also be presented as an argument for limited or refusal of critical care support [20], but the nature critical illness makes this position a challenging quagmire. ICU support can be uncomfortable, traumatic, and may prolong the dying process. This can be viewed as causing harm to the patient. However, not providing ICU support can also be viewed as the ultimate form of harm (i.e. failure to rescue) as it may directly or indirectly contribute to patient demise. Consequently, this argument is not convincing unless the surrogate decision-maker believes that allowing death to occur is an appropriate and acceptable way to end suffering. The result is that existing ethical constructs are often not effective tools to facilitate discussion and shared decision-making in the complex end-of-life discussions that involve conflicting opinions between health care providers and recipients. Moreover, during crises of ICU capacity, we believe this perspective is not appropriate to guide triage and/or bed rationing decisions in critically ill patients.

## Can survival and outcome be predicted?

Physicians need to be aware of their own biases and the potential for the self-fulfilling prophecy of perception of poor outcome directly contributing in patient death. In fact, two powerful predictors of outcome relate to perception: 1) the physician's belief that the patient would prefer not to receive advanced life support, and 2) the physician's prediction of low likelihood of survival to discharge from ICU. Unfortunately, physicians often assume rather than ask directly about a patient's preferences regarding end-of-life care [21,22]. Furthermore, despite the appearance of confidence in estimating prognosis, health care professionals are generally poor at subjectively predicting survival, functional outcome and/or quality of life for those with critical illness [3,23,24]. Data also suggest that nurses are commonly more pessimistic when compared with physicians regarding prognostication, in particular when ICU support is perceived as futile. Regrettably, this often contributes to high rates of moral distress, emotional exhaustion and burnout amongst critical care nurses [25-27]. Importantly, these conflicts may also be associated with worse patient outcomes, including increased mortality, increased length of stay and more frequent readmissions to ICU [28]. This has led some to suggest that perhaps physicians need "rose-colored glasses", a euphemism for a more positive outlook, rather than a crystal ball to predict future outcome [29].

Presently there are no validated tools that universally discriminate survivors from non-survivors of critical illness at ICU presentation and no tests of capacity to heal [30]. Age alone has not been shown to be a consistent predictor of outcome in critical illness. A systematic review demonstrated that while physicians outperformed scoring systems for prognosticating within the first 24 hours of ICU admission, both were only moderately accurate [31]. Unfortunately, attempts to standardize prognostication through the use of severity of illness scoring systems, such as the Acute Physiology and Chronic Health Evaluation (APACHE) score, have largely failed as these tools are designed for evaluation and comparison of large patient populations rather than predicting outcome in individual patients. It is interesting that the vast majority of surrogate decision-makers of critically ill patients want physicians to admit prognostic uncertainty, perhaps a reflection of more trust for the physician who admits human fallibility [32,33].

It should be noted that survivors of critical illness commonly state they would accept further ICU therapy should it be necessary, indicating that ICU care is perhaps not as unpleasant as either health care professionals believe or patients recall [34,35]. This implies there may be an important discrepancy between physician perception on both the utility and experience of ICU support and the reality experienced by patients. Furthermore, the prognostic focus for patients with critical illness has traditionally centered on short-term mortality; increasingly, however, additional patient-centered outcomes are being recognized as having equal or perhaps greater relevance for survivors of critical illness including functional capacity, neuro-cognitive impairment, disability, and quality of life [36]. As with predicting survival, our ability to prognosticate about the future of these outcomes for a large proportion of patients at the time of presentation remains largely untested.

## What is frailty?

Severity of illness scoring systems used in ICU (i.e. APACHE II) are largely dominated by acute physiologic derangements present at the time of admission, although some incorporate a limited assessment of confirmed advanced co-morbid illness. There is mounting evidence that physiologic reserve is an important aspect of the observed mortality for critically ill patients, as baseline functional status (i.e. disability) and pre-existing co-morbid disease have prognostic utility [37-40]. Disability is defined by difficulty or inability in performing activities essential for independent living and co-morbidity merely represents the coexistence of at least two separate pre-existing diagnosed illnesses [41].

**Figure 1:**
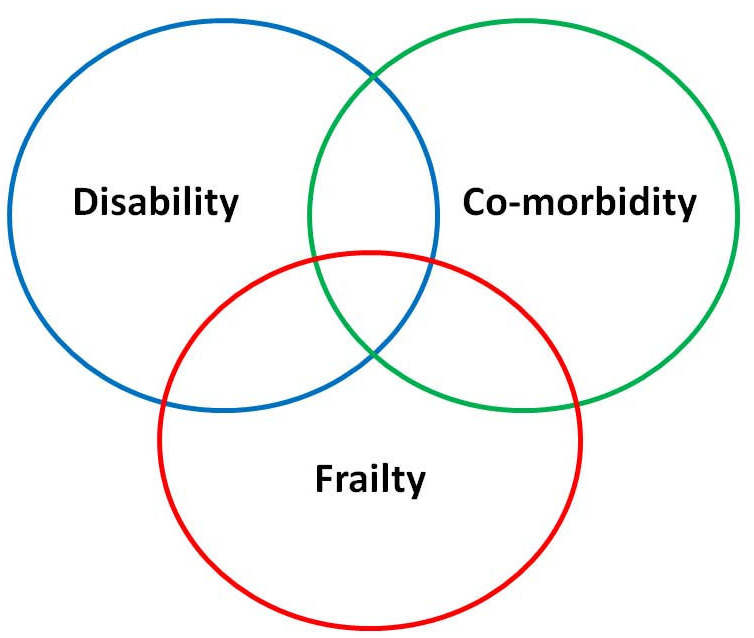
**Venn diagram showing the overlap and relationships between disability (defined by ≥ 1 ADL), co-morbidity and frailty (adapted from **[43]).

Gerontologists have recently defined the notion of "frailty" as a multidimensional syndrome characterized by the loss of reserve, where deficits accumulate that individually are reversible but collectively often represent an insurmountable burden of disease [42]. Frailty focuses on changes to mobility, muscle mass, nutritional status, strength and endurance. While frailty can have significant overlap with disability and co-morbidity, it is a distinct syndrome and is characterized by a vicious cycle of decreasing muscle mass, energy expenditure and malnutrition culminating in vulnerability to adverse events [43]. (see Figure 1)

Frailty is intimately correlated with the ageing process. Ageing has been associated with an increased pro-inflammatory response [44]. This pro-inflammatory state may provide a protective advantage during the reproductive years, however, it can also contribute to a low grade chronic inflammatory state in later years that exhausts the compensatory anti-inflammatory response [45]. Franseschi and colleagues have speculated that the ageing process of a species has a strong evolutionary basis and is able to respond to selection pressure to maintain ecological equilibrium. In this model, individual differences in longevity can be explained by differing capacities for adaptation and remodeling that have been selected at the population level [46]. In fact, genetic studies of centenarians have shown that the majority of polymorphisms involve anti-inflammatory genes, underscoring the importance of inflammation to ageing.

This concept has been further developed by Fried and colleagues in their explanation of the phenotype of frailty [43]. The critically ill patient, in many respects, is analogous to the frail geriatric patient, in that physiologic reserve is inadequate to maintain homeostasis. In fact, frailty correlates better with mortality in the elderly than chronologic age. Moreover, frailty has been shown to have an exponential relationship with mortality that ultimately results in "an avalanche-like destruction of the organism by the accumulation of deficits" [47,48]. Currently, validated scoring systems of frailty have only been evaluated in the elderly [41,43,49,50]. The impact of frailty on the clinical course and outcome of patients presenting with critical illness has yet to be investigated. However, we speculate that frailty applies across a broad spectrum of age when assessed in the context of critical illness and likely has important interaction with several factors including illness severity, co-morbid illness, biogenetics and the environment. As such, we believe frailty has particular relevance in critical illness. A global measure of an individual patient's frailty may serve as a more robust and consistent determinant for survival to hospital discharge and/or the capacity for sustaining reasonable independent function. Furthermore, we postulate that the degree and trajectory of ongoing loss of reserve leading to homeostatic imbalance prior to the onset critical illness may be a marker of the inability to heal from severe physiologic stressors that occur during critical illness. Prospective observational studies evaluating measures to predict survival and functional independence are urgently needed to test these hypotheses. Such data would also provide needed support and reinforcement to physicians confronted with decisions on the appropriateness of ICU support. Ideally, if frailty is proven to have value, such evidence could then be applied to guide ICU triage and decision-making at a health policy/societal level. Similarly, the inclusion of measures of frailty into cost-utility analyses would aid in identifying subgroups of ICU patients for whom ICU would be least likely to preserve quality-adjusted survival. Finally, we also believe, the simple assessment of frailty, assuming it is proven to have important prognostic value, would operate independent of ICU capacity crises, and could, theoretically at least, contribute to a more just and efficient use of resources.

## Communication at the bedside

Pragmatically, we suggest discussions regarding ICU triage and end-of-life care should initially focus on goals of therapy, incorporating an assessment of frailty, rather than on specific treatments (such as cardiopulmonary resuscitation) or on the futility thereof. To explore frailty, health care providers necessarily must engage family about objective measures of pre-existing functional status and quality of life to obtain an accurate representation of the individual. In contrast to discussions of futility, which invariably pit the health care provider against the family, inquiring about frailty requires dialogue and more open-ended questioning. Studies of family conferences have shown that the time families and surrogates spend talking correlates directly with family satisfaction and improved compliance with the treatment plan, and inversely with the risk of litigation [51,52].

## The future

These challenges regarding ICU support and end-of-life care represent exciting opportunities for physicians to explore their role in society. Perhaps an important step forward should be developing a more explicit segregation of the gatekeeper responsibilities of physicians as clinicians and as health care stewards. This may allow clinicians at the bedside to focus on their patients' well-being to the best of their ability, and engage in the process of shared decision-making. Physicians have a great deal of responsibility in the patient-physician interaction due to the power imbalance inherent in the relationship. They must recognize and honor the vulnerability of the patient and their surrogates, being mindful to not impose personal values and beliefs in the process of providing care. Greater responsibility for professionalism must be distinguished from greater authority to control choices, values and preferences. This may be seen as imposing unfair burden on physicians, but this asymmetry in responsibility is essential to protect the vulnerable from authoritarianism [53]. Simultaneously, they must not circumvent the responsibility to guide patients – uninformed choice is not synonymous with patient autonomy.

Concomitantly, explicit segregation of the role of health care steward would ameliorate some of the conflict of interest regarding resource allocation issues at the bedside. The natural ebb and flow in demand for ICU resources requires preemptive planning for surge capacity in times of high capacity, both on a local and global scale. The fundamental unit of the critical care resource is a "bed" in the ICU with its associated technological support and personnel. Locally, the responsibility of the steward would be to ensure that a plan for the next patient in need is continuously in place, minimizing therapeutic delays for new critically ill patients. This would include facilitation of overflow of critical care patients into other hospital areas such as the post-operative recovery areas, cancellation of elective surgeries, and transfer of individuals to other institutions. On a societal level, stewards would assume leadership roles and proactively assist hospital policymakers and government in developing reasonable thresholds for admission and discharge from ICU, supported by available evidence and based on a reasonable expectation of a non-trivial benefit. The concept of frailty has promise in the definition of non-trivial benefit, since frailty is related to failing function, and acceptable quality-adjusted functional outcome is as important as survival to many patients.

Simultaneously, critical care physicians must engage the public in the matter of health care reform and priority setting. As a profession, physicians need a strong body of leadership and a sense of unity for physician-driven health care reform to be a reality. Nationally and internationally recognized not-for-profit organizations, such as the Canadian Medical Association, or additional key stakeholders such as the Canadian Critical Care Society or the European Society of Intensive Care Medicine, should be amongst those who lead the dialogue, engage government and the public, and facilitate consensus and/or reform with respect to these sensitive issues. Ultimately, when capacity is consistently exceeded, decisions will have to be made regarding whether access to critical care becomes reasonably limited, more money is allocated or both. In the context of critical care support, reasonable limitations can be vague, provoke serious ethical dilemmas and directly conflict with the moral imperative of the "rule of rescue" for those identifiable patients faced with immediate peril [54,55]. Broadly, these decisions must be guided by what our society considers to be the inherent value of human life and the resultant financial burden society is willing to bear for the provision of modern public health care.

"Those who have the privilege to know have the duty to act."

Albert Einstein

## Abbreviations

APACHE: Acute Physiology and Chronic Health Evaluation; ICU: intensive care unit.

## Competing interests

The authors declare that they have no competing interests.

## Authors' contributions

Both authors contributed to the writing and critical revision of the manuscript.

## About the authors

Dr. McDermid is a consultant intensivist at the University of Alberta Hospital General Systems Intensive Care Unit and an Associate Clinical Professor with the Division of Critical Care Medicine, University of Alberta, Edmonton, Canada. He is interested in resident education and end-of-life care.

Dr. Bagshaw is a consultant intensivist at the University of Alberta Hospital General Systems Intensive Care Unit and an Assistant Professor with the Division of Critical Care Medicine, University of Alberta, Edmonton, Canada. His primary research interests consist of acute kidney injury and extracorporeal therapies in critically ill patients, Medical Emergency/Rapid Response Teams, and end-of-life care.
